# Supplementary material for: PopMAG: a Nextflow pipeline for population genetics analysis based on metagenome-assembled genomes
Source: Bioinform Adv. 2026 Jun 3;6(1):vbag150. doi: 10.1093/bioadv/vbag150 (PMC13268803; doi:10.1093/bioadv/vbag150)
Supplement: vbag150_Supplementary_Data [file vbag150_supplementary_data.pdf]

PopMAG: A Nextflow pipeline for population genetics analysis based on Metagenome-Assembled Genomes

Daniel Sabogal-Rodriguez[1,3], Alejandro Caro-Quintero[2,3]*

[1] Departamento de Ingeniería de Sistemas e Industrial, Universidad Nacional de Colombia, 111321, Bogotá, Colombia.

[2] Departamento de Biología, Facultad de Ciencias, Universidad Nacional de Colombia, 111321, Bogotá, Colombia.

[3] Max Planck Tandem Group in Holobionts, Universidad Nacional de Colombia, 111321, Bogotá, Colombia.

Supplementary figure S1: Bar chart displaying coverage distribution of MAG 35_bin.001 across 10 longitudinal cystic fibrosis lung metagenome samples, with proportional abundances shown above bars. The normalized Levins' index (Bn = 0.617) indicates a moderately generalist distribution, with the MAG present in all samples but at varying abundances. Samples are ordered by decreasing coverage.

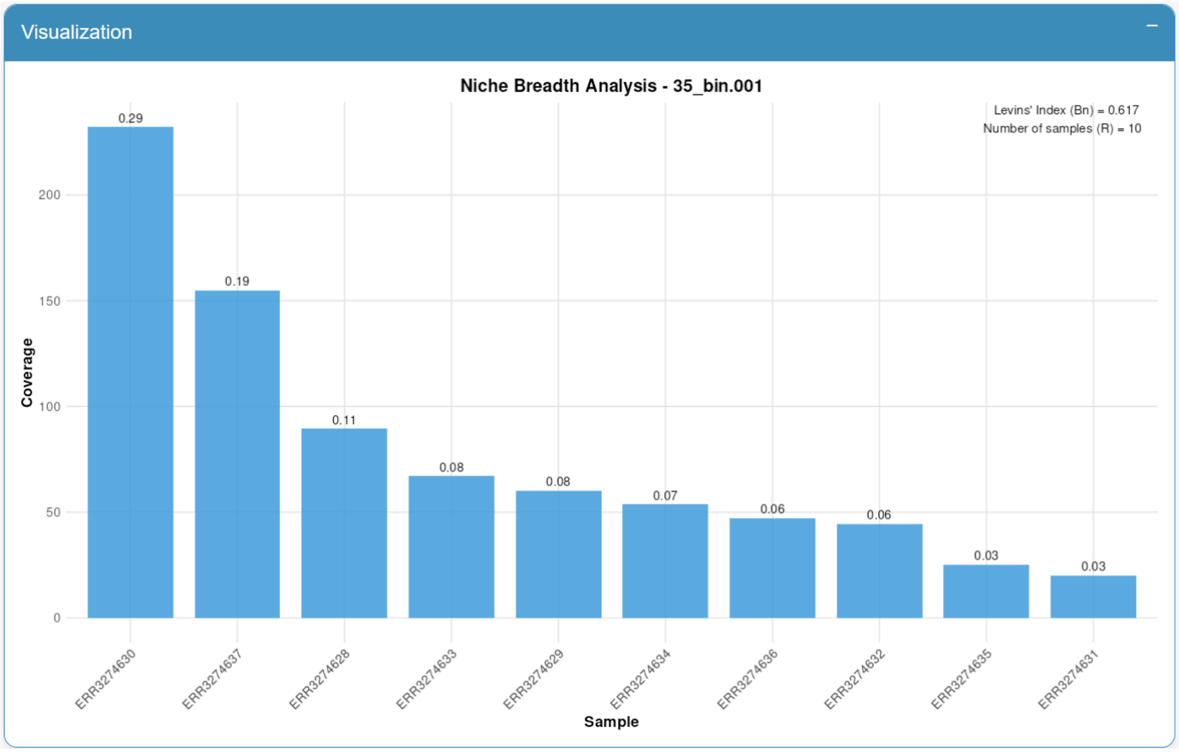

Supplementary figure S2: Bar chart displaying nucleotide diversity (π) for MAG 35_bin.001 across 10 longitudinal cystic fibrosis samples. The red line indicates 50× rarefied diversity to account for coverage variation. A marked decrease in diversity at sample ERR3274631 suggests a potential population bottleneck or selective event. Diversity values range from ~7×10^{-5} to ~6×10^{-4}.

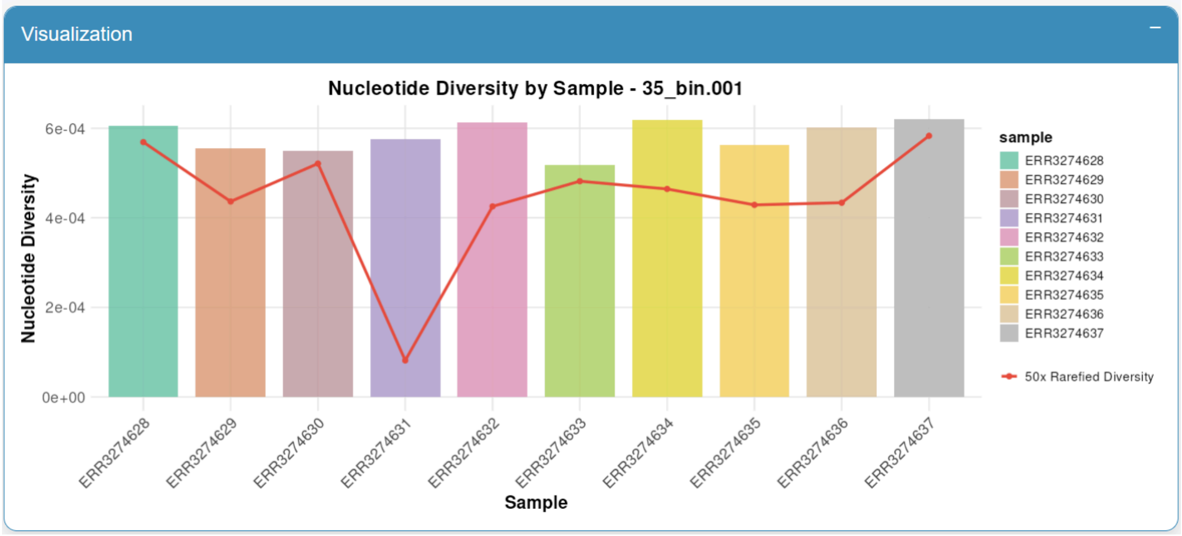

Supplementary figure S3: Scatter plots correlating nucleotide diversity (π) with clinical metadata: exacerbation status (left), C-reactive protein levels (center), and sampling day (right). Points are colored by sample ID and sized by coverage. Dashed lines show linear regression fits with 95% confidence intervals. This metadata-driven visualization enables exploration of associations between population genetic metrics and clinical covariates.

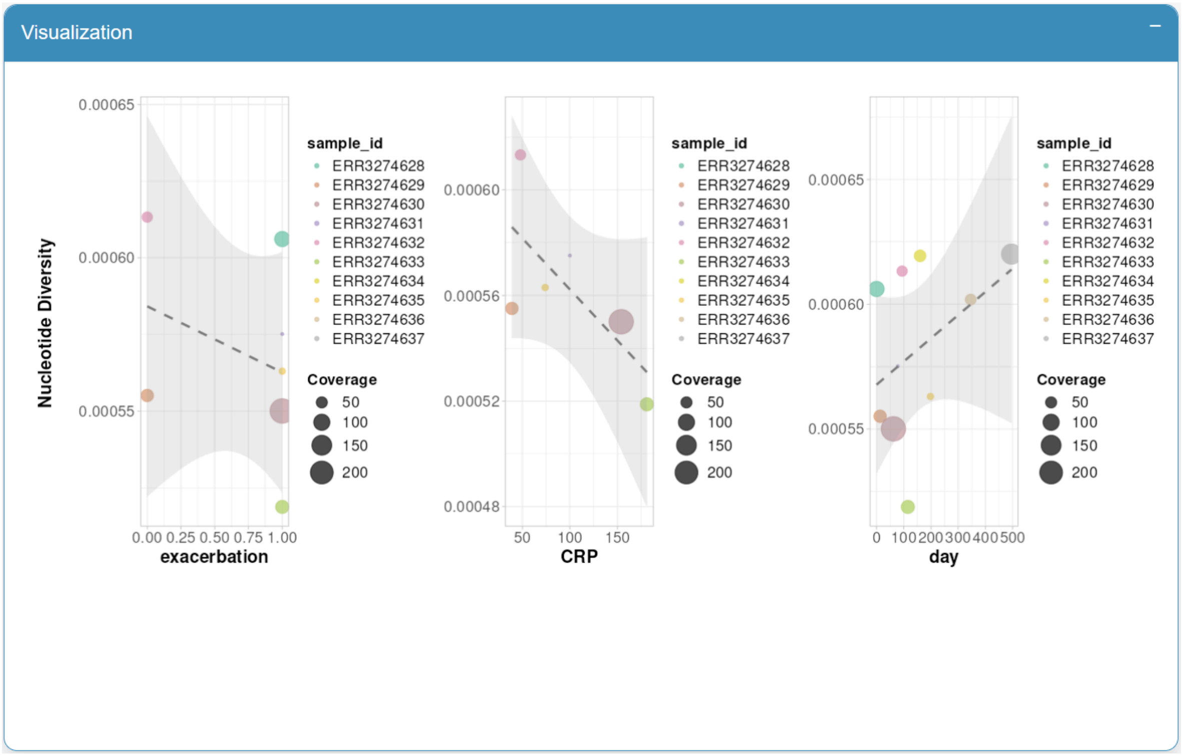

Supplementary figure S4: Heatmap displaying fixation indices (FST) between all sample pairs for MAG 35_bin.001 across longitudinal cystic fibrosis samples. Color scale ranges from yellow (FST ≈ 0, low differentiation) to red (FST ≈ 0.5, high differentiation). Elevated FST values involving ERR3274630 (up to 0.307) indicate population structure changes at this time point, potentially reflecting strain replacement or selective sweeps.

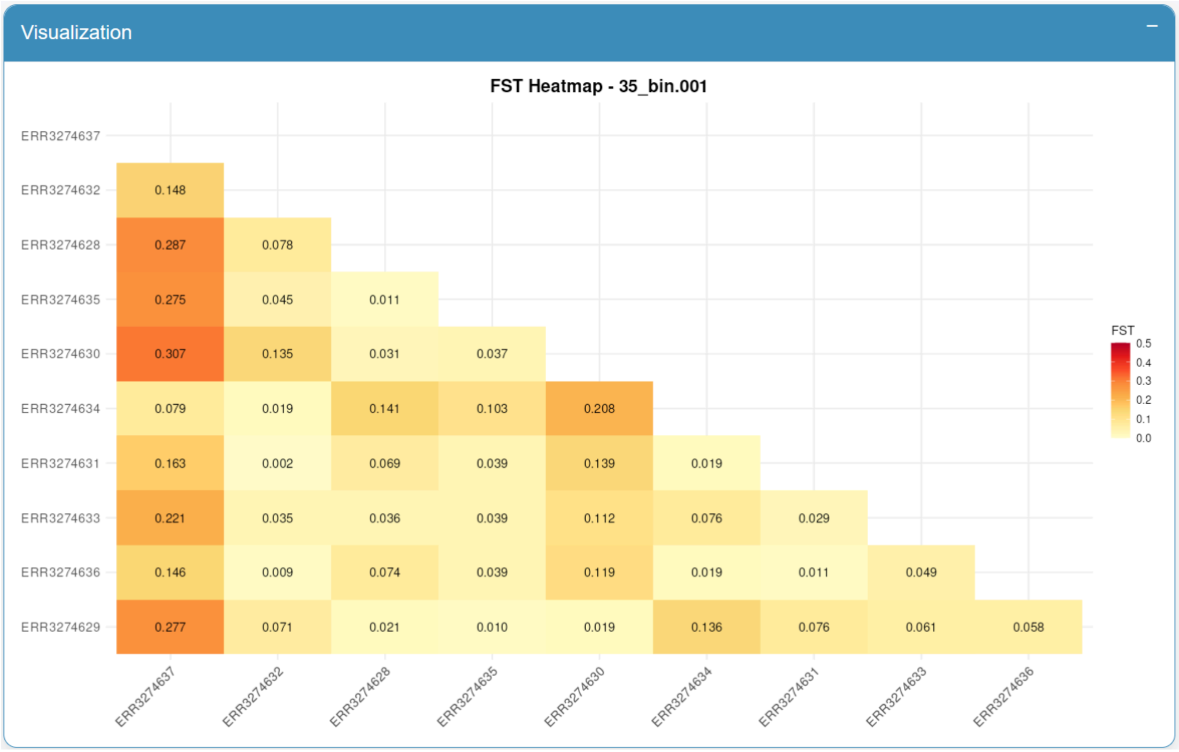

Supplementary figure S5: Histogram showing the distribution of gene-level pN/pS ratios for MAG 35_bin.001 across all samples. The dashed red line indicates neutral selection (pN/pS = 1). The strong left-skew (majority of genes with pN/pS < 0.1) is consistent with purifying selection, while rare genes with pN/pS > 1 represent candidates under positive selection.

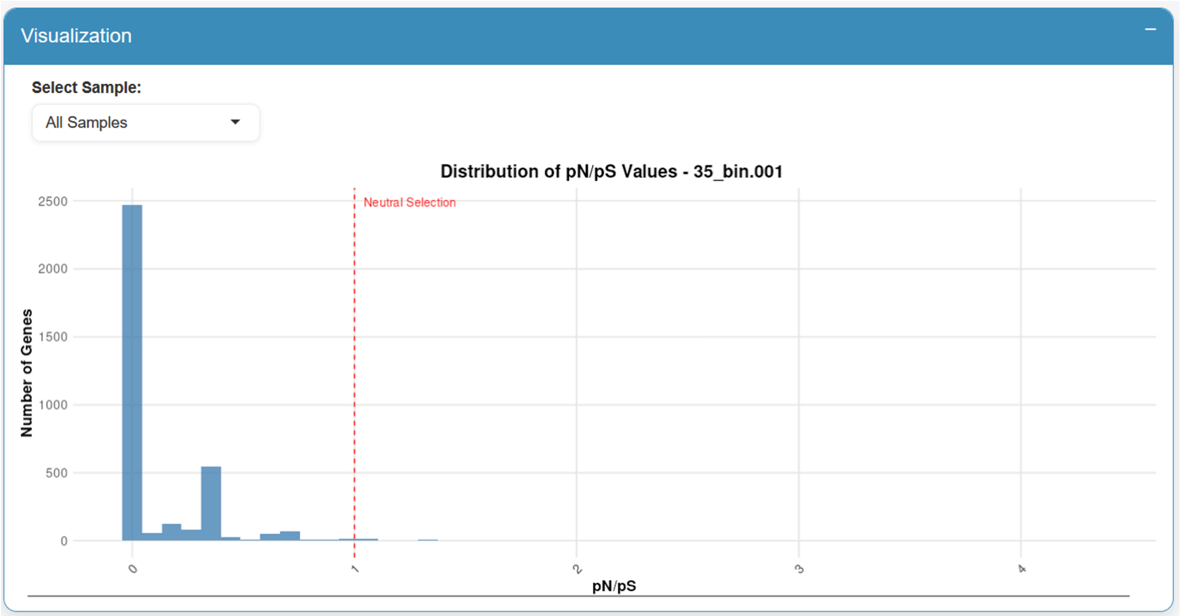

Supplementary figure S6: Interactive table displaying per-gene population genetics metrics including pN/pS, nucleotide diversity, coverage, and functional annotations. The search and filter functionality enables targeted exploration of genes of interest. Here, the MexA (AcrA) efflux pump gene shows elevated pN/pS (2.1) in sample ERR3274637 compared to earlier time points (pN/pS = 0.0), consistent with positive selection potentially driven by antibiotic pressure. This gene has 1,152 bp in length (~384 codons), with 6 nonsynonymous and 1 synonymous SNVs.

Data Table

Search:

Sample	Product	pN/pS	Nucleotide Diversity	Coverage	Breadth	Best Hit	EC Number
All	iit MexA	All	All	All	All	All	All
ERR3274637	multidrug efflux RND transporter periplasmic adaptor subunit MexA	2.1	0.0	162.6	1.0	NF033834.1	
ERR3274628	multidrug efflux RND transporter periplasmic adaptor subunit MexA		0.0	109.3	1.0	NF033834.1	
ERR3274629	multidrug efflux RND transporter periplasmic adaptor subunit MexA		0.0	74.4	1.0	NF033834.1	
ERR3274630	multidrug efflux RND transporter periplasmic adaptor subunit MexA		0.0	266.3	1.0	NF033834.1	

Supplementary table 1: ENA accession numbers and relevant metadata for the metagenomic samples used in the case study section.

Patient	Day of treatment	FEV (%)	CRP (mg/ml)	Accession number
CFR11	0	NA	NA	ERR3274628
CFR11	13	NA	39	ERR3274629
CFR11	62	34	154	ERR3274630
CFR11	78	NA	100	ERR3274631
CFR11	94	NA	48	ERR3274632
CFR11	115	NA	181	ERR3274633
CFR11	160	32	NA	ERR3274634
CFR11	198	NA	74	ERR3274635
CFR11	346	NA	NA	ERR3274636
CFR11	496	NA	NA	ERR3274637
